# Distance-AF Multimer: A Multi-Step Approach for Protein Complex Structure Modeling with User-Defined Distance Constraints

**DOI:** 10.64898/2026.10.14.759232

**Published:** 2026-10-18

**Authors:** Kai Ling, Yuanyuan Zhang, Zicong Zhang, Tsukasa Nakamura, Daisuke Kihara

## Abstract

The three-dimensional structure of proteins provides essential insight into the molecular mechanisms underlying their function. Many biological activities are mediated through protein interactions, making accurate structural determination of protein complexes important. Despite significant advances in computational protein structure prediction, there are still many cases where protein complexes are not correctly assembled. In particular, there are cases where experimental data, such as distances between residue pairs in interacting proteins are available, yet existing prediction methods fail to produce models consistent with these data. Here, we present Distance-AF Multimer, a method that guides and refines AlphaFold-Multimer docking predictions using user-provided residue pair distance information. This approach extends our previously successful data-guided single-protein modeling method, Distance-AF. Distance-AF Multimer operates in a two-step process, starting from multiple initial conformations and iteratively refining them to satisfy the provided distance constraints. We evaluated Distance-AF Multimer on protein-complex benchmarks spanning different chain counts and docking errors, demonstrating competitive or superior performance compared to other existing deep-learning-based structure prediction methods. In addition to dimer cases, we also demonstrated the potential of Distance-AF Multimer for large-scale protein assemblies containing multiple chains, including those with more than 5 chains.

**Significance Statement:**

Proteins often function as complexes, but predicting how their components fit together remains difficult. Distance-AF Multimer uses distance constraints between residues to improve the arrangement of protein chains in predicted structures. This approach offers a practical way to combine computational predictions with available structural information, helping researchers study protein interactions, peptide binding, and larger assemblies when detailed experimental structures are unavailable.

